# Thyroid gland changes in patients with acromegaly

**DOI:** 10.20945/2359-3997000000247

**Published:** 2020-06-05

**Authors:** Emil Natchev, Silvia Vandeva, Roussanka Kovatcheva, Georgi Kirilov, Krasimir Kalinov, Sabina Zacharieva

**Affiliations:** 1 Department of Endocrinology Medical University Sofia Bulgaria Department of Endocrinology, Medical University, Sofia, Bulgaria; 2 New Bulgarian University Sofia Bulgaria New Bulgarian University, Sofia, Bulgaria

**Keywords:** Acromegaly, thyroid dysfunction, goiter, GH, IGF-1, thyroid ultrasonography

## Abstract

**Objective:**

Acromegaly is characterized by high neoplastic morbidity as a side effect of growth hormone (GH) hypersecretion. Increased incidence of goiter, thyroid carcinoma, and thyroid dysfunction is also reported. The aim of the present study was to find the prevalence of thyroid dysfunction and goiter in patients with acromegaly and determine its relationship to disease activity, disease duration, and the presence of secondary hypothyroidism.

**Subjects and methods:**

In a cross-sectional study of the period 2008-2012 were included 146 patients with acromegaly (56 men, 90 women) of mean age 50.3 ± 12.4 years. Acromegaly disease activity and thyroid function were evaluated in all patients. Thyroid ultrasonography was performed to calculate thyroid volume and detect the presence of nodular goiter.

**Results:**

Ninety-one patients were determined to have an active disease, and 55, a controlled disease. The mean thyroid volume in patients without previous thyroid surgery was 37.6 ± 38.8 mL. According to disease activity, thyroid volume was significantly higher in patients with active disease (38.5 ± 45.4 mL vs. 27.2 ± 18.4 mL, p = 0.036). A weak positive correlation was found between thyroid volume and insulin-like growth factor 1 (IGF-1) in the whole group and in females (R = 0.218; p = 0.013, and R = 0.238; p = 0.037, respectively). There was no significant correlation of thyroid volume with disease duration and GH level in the whole group and in both sexes. The patients with secondary hypothyroidism had twofold smaller thyroid volume, relative to the rest of the group. The prevalence of thyroid dysfunction was 39%, with a female to male percentage ratio of 1.73. Goiter was diagnosed in 87% of patients, including diffuse goiter (17.1%) and nodular (69.9%), with no significant difference between patients with active and controlled disease or the presence of secondary hypothyroidism.

**Conclusions:**

Thyroid volume in patients with acromegaly depends on disease activity and the presence of secondary hypothyroidism as a complication. The increased prevalence of nodular goiter determines the need of regular ultrasound thyroid evaluation in the follow-up of patients with acromegaly. Arch Endocrinol Metab. 2020;64(3):269-75

## INTRODUCTION

Acromegaly is a clinical syndrome produced by growth hormone (GH) hypersecretion, which is, in most cases, caused by pituitary adenoma. One of the main characteristics of this syndrome is increased neoplastic formation, including nodular goiter ( 1 - 3 ). The frequency of goiter is high among acromegalic patients ( 4 - 8 ). In 1932, Rolleston and cols. first suggested a relationship between acromegaly and the pathogenesis of goiter ( 9 ). Goiter in patients with acromegaly is predominantly nodular and there is also an increased prevalence of thyroid cancer ( 4 , 6 - 8 , 10 ). Euthyroid nodular goiter is more frequent, while the frequency of the toxic one is around 14% ( 5 , 6 , 11 , 12 ). The prevalence of autoimmune thyroid diseases among acromegalic patients is comparable to that of the general population ( 13 ). Presumably, in patients with acromegaly, the pathogenesis of goiter is related mainly to an increased level of insulin-like growth factor-1 (IGF-1), which binds to its specific receptor, expressed in thyrocytes ( 14 ). By contrast, another important factor associated with goiter formation, thyroid stimulating hormone (TSH), is suppressed or absent in some patients with acromegaly. The main reason for that is secondary hypothyroidism, which is caused by compression by the adenoma, pituitary surgery, or irradiation ( 15 , 16 ).

The aim of the present study was to find the prevalence of thyroid dysfunction and goiter in patients with acromegaly, and determine its relationship with disease activity, disease duration, and the presence of secondary hypothyroidism.

## SUBJECTS AND METHODS

### Patients

In this cross-sectional study of the period 2008-2012 were included 146 patients with acromegaly (56 men, 90 women), mean age of 50.3 ± 12.4 years. The study protocol was approved by the local ethics committee. Each subject was informed about the study protocol and signed informed consent forms. All patients were euthyroid, including those with thyroid dysfunction, as they were under appropriate medical treatment. According to disease activity, the patients were divided into two groups: group 1 consisted of patients with active acromegaly (n = 91); and group 2 consisted of patients in remission (n = 55). Each group was divided according to the patients’ gender. The characteristics of the groups, including the levels of GH, IGF-1, and treatment, are described in Table 1 .

**Table 1:** Characteristics of patients groups according to activity of acromegaly and treatment

Parameters	Active acromegaly		Disease control	
	
	Males (n = 35)	Females (n = 56)	Males (n = 21)	Females (n = 34)
GH (mean ± SD)	29.19 ± 28.98	19.8 ± 24.7	2.35 ± 3.46	1.99 ± 1.69
IGF1 (mean ± SD)	78.8 ± 40.88	64.66 ± 33.39	23.71 ± 10.89	21.19 ± 12.39
IGF1/ ULN (mean ± SD)	2.04 ± 0.99	1.71 ± 0.92	0.67 ± 0.3	0.59 ± 0.33
Treatment method (n)	23	42	21	34
TSA	7	11	14	16
TSA+RT	0	5	4	8
DA	3	2	1	0
TSA+DA	5	15	2	4
TSA+SSA	2	0	0	0
TSA+DA+SSA	1	1	0	0
TSA+RT+ DA	1	6	0	6
TSA+RT+SSA	3	2	0	0
TSA+RT+DA+SSA	1	0	0	0

n: number of patients; GH: growth hormone, measured in mIU/L, conversion factor to ng/ml is 3; IGF-1: insulin-like growth factor 1, measured in nmol/l; ULN: upper limit of normal; TSA: transsphenoidal adenomectomy; RT: radiotherapy; DA: dopamine agonist; SSA: somatostatin analogs.

The disease onset was identified using the data from patients’ medical history, information from their close relatives, and old photographs. The duration of acromegaly was defined as the time between the disease onset and the last follow-up. For the definition of disease activity and disease control, we followed the consensus guidelines of 2010, using the levels of both IGF-1 and GH ( 17 ).

## METHODS

### Laboratory tests

All hormonal assays were performed in a certified and centralized laboratory. Serum GH was determined by a commercial kit DELFIA (Perkin Elmer Life and Analytical Sciences; Wallac Oy, Finland), with sensitivity of < 0.03 mIU/L. The intra- and inter-assay coefficients of variation were 3.9% and 5.0%, respectively.

Serum IGF-I was measured by high sensitive immunoradiometric assay (Immunotech; Beckman Coulter Co., France), with sensitivity of < 0.26 nmol/L. The intra- and inter-assay coefficients of variation were 6.3% and 6.8%, respectively.

Serum TSH was measured by immunoradiometric assay (THERMO scientific/BRAHMS, Germany), with reference ranges of 0.3-4.0 mIU/l. The sensitivity was 0.02 mIU/l, with intra- and inter- assay coefficients of variation of 2.5% and 4.1% respectively.

Serum FT4 was measured by radioimmunometric assay (THERMO scientific/BRAHMS, Germany), with reference ranges of 9-24 pmol/L. The sensitivity was 1.25 pmol/L, with intra- and inter- assay coefficients of variation of 3.4% and 5.1% respectively.

Anti-thyroid peroxidase antibodies (Anti-TPO) was measured by radioimmunometric assay (THERMO scientific/BRAHMS, Germany). Values greater than 60 U/mL are regarded as positive. The functional assay sensitivity has been assessed as being < 50 U/mL, with intra- and inter- assay coefficients of variation of 4.5% and 9.9%, respectively.

### Ultrasonography

In all participants, thyroid ultrasonography was performed by the same physician (E.N.), with Toshiba ECCOCEE, SSA – 340A and 10 MHz linear transducer. Thyroid volume was calculated as a sum of both lobes and isthmus volumes, using the formula for an ellipsoid (π/٦ *. a.b.c* ), where *a* is the longitudinal diameter, *b* is the transversal diameter, and *c* is the antero-posterior diameter. The presence of goiter was determined if thyroid volume was > 24.31 mL for men and > 18.78 mL for women. The detection of thyroid nodule/s over 5 mm in diameter was classified as nodular goiter ( 18 ).

### Statistical methods

The following statistical methods were used to present and summarize the results and conclusions ( 19 ):

1. Descriptive methods. Categorical data were presented as number of patients and respective percentages. Metric data was presented as a mean ± standard deviation (SD), or median, and highest and lowest value.
2. Methods of statistical inference. To compare the means of two independent groups, we used the Student’s t-test. Exact – χ^2^test was used to compare non-metrical data. Correlation between variables was evaluated by Pearson’s correlation coefficient.

A P value < 0.05 was considered statistically significant.

## RESULTS AND DISCUSSION

### Results

Thyroid dysfunction was present in 93.7% (15/16) of patients with previous thyroid surgery and in 32.3% (42/130) of patients with no previous surgery. The overall prevalence of thyroid dysfunction was 39% (57/146), and the calculated female to male percentage ratio was 1.73. More patients’ characteristics and thyroid dysfunction distribution are shown in Table 2 .

**Table 2:** Demographic characteristics and thyroid dysfunction distribution in patients with acromegaly

Characteristic	Value
Patients	
Sex*	
Female	90 (61.6%)
Male	56 (38.4%)
Age (y)	50.3 ± 12.4 (21-78)
BMI (kg/m^2^)	29.6 ± 5.2 (20-45)
Duration of acromegaly (y)	16.7 ± 7.5 (8-38)
Previous thyroid surgery*	
No	130 (89%)
Yes	16 (11%)
Thyroid dysfunction*	
Primary hypothyroidism	20 (13.7%)
Secondary hypothyroidism	22 (15.1%)
Primary and secondary hypothyroidism	7 (4.8%)
Hyperthyroidism	8 (5.5%)

Note. Unless otherwise indicated, data are means ± standard deviation, with the range in parentheses.

* Data are the number of patients, with the percentage in parentheses.

* The P value is obtained using Student’s t-test, comparing the means between females and males.

Autoimmune thyroid disease was found in 17.1% of all patients. The prevalence in in females was 24.4%, while in males, it was 5.3%. In patients with secondary hypothyroidism, the frequency of thyroid autoimmunity was comparable to the rest of the group – 17.2% (5/29) vs. 17.1% (20/117), p = 1.0, and to those with primary hypothyroidism – 17.2% (5/29) vs. 20% (4/20), p = 1.0.

The mean thyroid volume was 37.6 ± 38.8 mL in patients without previous thyroid surgery (n=130) and no significant difference was found between males (n = 53) and females (n = 77) – 45.8 ± 41.5 vs. 32.0 ± 36.0 ml, respectively, p = 0.053.

**Figure 1:**
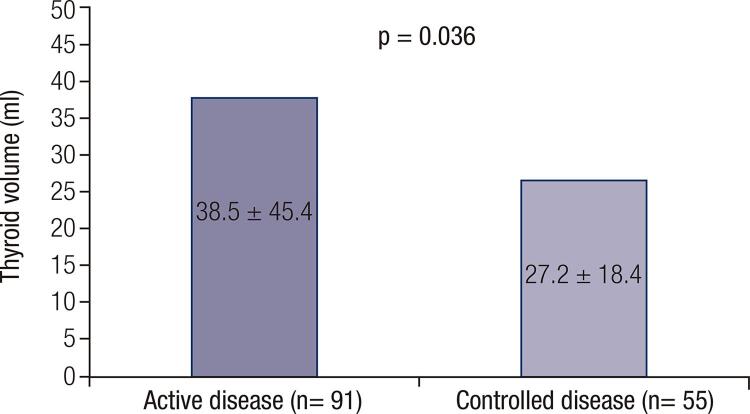
Comparison of thyroid volume between patients with active and controlled acromegaly Note. Data are means ± standard deviation. The P value is obtained using Student’s t-test.

In patients with secondary hypothyroidism, thyroid volume was significantly lower than in the rest of the non-operated patients ( Table 3 ). The significance was present in both sexes. According to disease activity, the analysis of thyroid volume showed significantly higher values in patients with active disease, compared to patients with controlled disease ( Figure 1 ). A weak positive correlation was found between thyroid volume and IGF-1 in the whole group of non-operated patients (n = 130) and in female patients (n = 77) – R = 0.218; p = 0.013, and R = 0.238; p = 0.037, respectively. No correlation was found in men (n = 53) – R = 0.156; p = 0.266. When IGF-1 values were expressed as a ratio between the current IGF-1 value and the upper limit of normal (ULN), similar figures were observed – R = 0.252; p = 0.004 for the group of non-operated patients; R = 0.287; p = 0.011 for females and R = 0.183; p = 0.19 for males. There was no significant correlation of thyroid volume with disease duration and GH level in the whole group – R = -0.036; p = 0.682, and R = 0.114; p = 0.195, respectively and in both sexes.

**Table 3:** Comparison of thyroid volume of patients with secondary hypothyroidism and the rest of the group

Patients	Thyroid gland volume (mL)		P value

	Patients with secondary hypothyroidism	Patients without secondary hypothyroidism	
Females	17.9 ± 4.4 (13)	34.8 ± 38.8 (64)	0.001
Males	28.6 ± 13.2 (8)	48.8 ± 44.1 (45)	0.017

Note. Data are means ± standard deviation, with the number of patients in parentheses. All patients are non-operated. The P value is obtained using Student’s t-test.

Goiter was diagnosed in 87% of our patients, including diffuse goiter (17.1%) and nodular goiter (69.9%). According to disease activity, the distribution of goiter was comparable and there was no significant difference in goiter prevalence between patients with active disease and disease control ( Table 4 ).

**Table 4:** Prevalence of goiter in patients with active acromegaly and disease control

Goiter	Active acromegaly (n = 91)	Disease control (n = 55)	P value
No goiter	11 (12.1%)	8 (14.5%)	0.8
Diffuse	19 (20.9%)	6 (10.9%)	0.173
Nodular	61 (67.0%)	41 (74.5%)	0.359

Note. Data are the number of patients, with the percentage in parentheses.

The P value is obtained using exact – χ^2^ test.

Among patients with previous surgery for nodular goiter (n = 16), 3 (18.7%) were diagnosed with papillary thyroid cancer on histology. Referring to all patients with nodular goiter (102) and to the whole group (146), the cases with thyroid cancer in our study are 2.94 % and 2.05%, respectively.

We also analyzed the prevalence of nodular goiter in patients with thyroid dysfunction. There was no significant difference in the frequency of nodular goiter in patients with secondary hypothyroidism and the rest of the group – 75.9% (22/29) *vs.* 68.4% (80/117), p = 0.504.

## DISCUSSION

The main focus of the study is morphological changes in the thyroid gland, particularly nodular goiter and thyroid gland volume. The high frequency of goiter, along with prevalence of the nodular type, found in our study, suggests a major role of hypersomatotropism in thyroid tumorigenesis, as also reported in other studies ( 5 , 16 , 20 , 21 ). This may be explained by the effect of increased IGF-1, both in the serum and locally, in the thyroid gland, or by the direct effect of GH ( 22 ). This hypothesis is supported by the absence of a significant difference in nodular goiter prevalence between patients with secondary hypothyroidism and the rest of the study group. Even more relevant, nodular goiter is not related to disease activity, as shown in our results. The absence of a statistically significant difference in the prevalence of nodular goiter between patients with secondary hypothyroidism and the rest of the study group suggests that TSH plays a less important role in thyroid tumorigenesis.

The role of IGF-1 in increased thyroid gland volume in patients with acromegaly has been confirmed by numerous studies ( 15 , 16 , 23 ). Continuous stimulation of thyrocytes by GH and IGF-1 results in an increase of thyroid volume ( 24 ). Studies with animal models show a synergic effect of IGF-1 and TSH on rat thyroid cells’ culture ( 25 ). Volzke and cols. have demonstrated the correlation of IGF-1 levels and thyroid volume in a population-based study ( 26 ). Brzozowska and cols. have found a similar association in children with normal ioduria ( 27 ). However, other authors do not reveal a correlation between the volume of the thyroid gland and the levels of GH and IGF-1 ( 5 , 7 ). Some studies show a decrease in thyroid volume, by up to 25%, after normalization of IGF-1, achieved through successful surgical removal of somatotropinoma, or by medical treatment with somatostatin analogs ( 6 , 15 , 16 ). Nevertheless, the independent influence of somatostatin analogs on thyroid volume should also be taken into consideration, as their receptors have been found in the thyroid tissue ( 15 , 25 , 28 ). In our study, patients with active acromegaly had significantly larger thyroid volume, relative to patients with controlled disease. This correlation was found in the whole group of patients and in females, but not in males. We found no significant correlation between thyroid volume and GH in any of the groups. Similarly, no correlation was demonstrated between thyroid volume and disease duration, contrary to fndings reported by other authors ( 15 , 29 ).

The role of TSH in the pathogenesis of goiter is well-established. Moreover, TSH was shown to potentiate the effect of IGF-1 on thyrocytes in a study using cell lines ( 30 ). This has also been confirmed by the small volume of the thyroid gland in hypopituitary patients, even among those who have undergone GH replacement therapy ( 31 ). According to some authors, the effect of TSH on goiter formation is expressed in the first years of acromegaly, while in the next stage, thyroid tumorigenesis becomes independent, or at least less dependent on TSH ( 15 , 16 , 31 ). In our study, patients with secondary hypothyroidism had twofold smaller thyroid volume, relative to the rest of the group. The probable influence of thyroid autoimmunity on thyroid volume was not confirmed by our data of similar frequency of autoimmune disorders in both groups. This finding also emphasizes TSH as an important factor in goiter pathogenesis in patients with acromegaly.

The predominant thyroid dysfunction in our study was hypothyroidism – primary and secondary, found in 33.6% of patients. Similar results, with a prevalence of about 25%, have been reported by other authors ( 7 ). We have a larger proportion of patients with secondary hypothyroidism, due to the higher historical rate of reoperations and radiotherapy, which may be attributed to the prior unavailability of medical treatment with somatostatin analogs and/or pegvisomant. The prevalence of hyperthyroidism was 5.5%, which is comparable to those reported by other authors ( 12 , 32 ). The frequency of thyroid autoimmunity found in our study (17.1% for the whole group; 24.4% of females and 5.3% of males) is comparable to the general Bulgarian population (23% of females and 10 % of males) ( 33 ). The female to male percentage ratio of 1.7 in our patients with thyroid dysfunction is lower than that in the general population, which provides additional confirmation of the role of IGF-1 and GH in thyroid growth and thyroid function ( 34 ).

Acromegaly is associated with a higher risk of tumorigenesis ( 10 , 35 , 36 ). Patients with active acromegaly have up to four times higher risk of developing neoplasia. According to the literature, the prevalence of thyroid carcinoma is about 3-7% of patients, represented mostly by the papillary variant ( 4 - 6 , 36 - 39 ). IGF-1 stimulates the proliferation of cancer cells, neoplastic angiogenesis, and metastasis ( 8 , 40 - 42 ). The direct effect of GH on tumorigenesis has also been discussed ( 22 ). Rogozinski et al. report 11% prevalence of thyroid carcinoma in a group of 34 patients with acromegaly, diagnosed by cytology and histopathology ( 20 ). The main indications for thyroid surgery in our group were goiter size, symptoms of compression, recurrent hyperthyroidism. The frequency of thyroid carcinoma in patients with thyroid surgery was 18.7 %, which is 3 times higher than in patients without acromegaly, operated on for nodular goiter in the same center and over the same period of time (6.3%). Referring to the entire group of patients with acromegaly, thyroid carcinoma presents about 2.05% of the time, representing 2.94% of patients with nodular goiter. Although our results are based on a low percentage of patients who have had surgery (11%), they are similar to those reported by Reverter et al., who found thyroid cancer in 3.3% of patients with thyroid nodules, corresponding to 1.6% of the whole group of patients with acromegaly ( 43 )

The power of our study lies in the significant number of patients with acromegaly, diagnosed and treated by the same protocol in a single tertiary center. Moreover, the thyroid ultrasound was performed and analyzed by the same physician. The study has some limitations, deriving from its cross-sectional design; we foresee the presentation of a longitudinal follow-up of our patients in a future study. Furthermore, the patients in our study with nodular goiter were not subjected systematically to fine needle aspiration biopsy, to ensure a more reliable selection for surgery. Another limitation of the study is its lack of control group.

In conclusion, the most frequent thyroid dysfunction in our study was secondary hypothyroidism, followed by primary hypothyroidism and hyperthyroidism. Thyroid volume in patients with acromegaly depends on disease activity and the presence of secondary hypothyroidism as a complication. The increased prevalence of nodular goiter determines the need for regular ultrasound thyroid evaluation in the follow-up of patients with acromegaly.
